# Neoadjuvant pyrotinib plus trastuzumab and chemotherapy for HER2-positive breast cancer: a prospective cohort study

**DOI:** 10.1186/s12957-023-03266-5

**Published:** 2023-12-19

**Authors:** Lu Liu, Mingzhi Zhu, Yanyan Wang, Muhan Li, Yuanting Gu

**Affiliations:** 1 Department of Breast Surgery, The First Affiliated Hospital of Zhengzhou University, 1 Jianshe East Road, Zhengzhou, 450052 Henan China; 2 Gastroenterology and Hepatology, The First Affiliated Hospital of Zhengzhou University, 1 Jianshe East Road, Zhengzhou, Henan 450052 China

**Keywords:** Pyrotinib, HER2, Breast cancer, Neoadjuvant therapy, p53

## Abstract

**Background:**

This prospective study aims to investigate the efficacy and safety of pyrotinib (P) combined with 4 cycles of epirubicin and cyclophosphamide followed by 4 cycles of taxane and trastuzumab (P + EC-TH) regimen as neoadjuvant therapy for human epidermal growth factor receptor 2 (HER2) positive breast cancer and to investigate the predictive value of p53, p63, and epidermal growth factor receptor (EGFR) status for neoadjuvant efficacy.

**Methods:**

A total of 138 HER2-positive breast cancer patients who received neoadjuvant therapy and underwent surgery were included. Case group: 55 patients received P + EC-TH regimen. Control group: 83 patients received EC-TH regimen. The chi-square test, Fisher’s exact test, and logistic regression analysis were applied. The primary endpoint was total pathologic complete response (tpCR), and the secondary endpoints were breast pathologic complete response (bpCR), overall response rate (ORR), and adverse events (AEs).

**Results:**

In the case group, the tpCR rate was 63.64% (35/55), the bpCR rate was 69.09% (38/55), and the ORR was 100.00% (55/55). In the control group, the tpCR rate was 39.76% (33/83), the bpCR rate was 44.58% (37/83), and the ORR was 95.18% (79/83). The case group had significantly higher tpCR and bpCR rates than those of the control group (*P* < 0.05), but there was no significant difference in ORR (*P* > 0.05). The tpCR was associated with the status of estrogen receptor (ER), progesterone receptor (PR), and androgen receptor (AR), and the patients with any negative ER, PR, AR, or combined, were more likely to achieve tpCR than those with positive results (*P* < 0.05). The p53-positive patients were more likely to achieve tpCR and bpCR than p53-negative patients (*P* < 0.05). The incidence of hypokalemia and diarrhea in the case group was higher than that in the control group (*P* < 0.05). The AEs developed were all manageable, and no treatment-related death occurred.

**Conclusion:**

The efficacy and safety of the P + EC-TH regimen were verified by this study. The HER2-positive breast cancer patients treated with the EC-TH neoadjuvant regimen were more likely to achieve tpCR or bpCR if pyrotinib was administered simultaneously.

## Background

In recent years, the incidence of breast cancer in women has been increasing [1]. Breast cancer is the leading cause of newly diagnosed cancer cases among American women based on data from 2023 and is still on the rise. The death rate of breast cancer in America is the second highest among all female cancers, posing a major threat to women’s health [2]. There are a variety of treatments for breast cancer, and neoadjuvant chemotherapy (NAC) is one of them for surgical operable patients. Many clinical trials have shown that preoperative NAC is as effective as postoperative adjuvant chemotherapy. It increases the proportion of patients who can undergo breast-conserving surgeries and reduces the need for axillary lymph node dissection (ALND) [3, 4]. In addition, NAC provides information on tumor response in vivo and is widely used in clinical practice. Reaching a pathologic complete response (pCR) after NAC indicates a better long-term prognosis [5]; therefore, it is particularly important to explore a regimen with a high pCR rate. NAC with anthracyclines and cyclophosphamide followed by taxanes has been widely used in clinical practice, whose curative effect is definite [6, 7]. Epirubicin is one of the representative drugs of anthracyclines, which can reduce cardiac toxicity during chemotherapy compared with traditional doxorubicin. Human epidermal growth factor receptor 2 (HER2)-positive breast cancer is a special subtype of breast cancer, accounting for 15% to 25% of all breast cancers [8]. Compared with other types of breast cancer, it is more aggressive, easy to metastasize in the early stage, and has a poor long-term prognosis [9]. Medical therapies based on anti-HER2 therapy have become the cornerstone of treatment for HER2-positive early breast cancer. The use of trastuzumab combined with chemotherapy in neoadjuvant therapy of HER2-positive patients has a higher pCR rate than that of chemotherapy alone, which lays the cornerstone position for trastuzumab in neoadjuvant therapy of HER2-positive breast cancer patients [10]. However, resistance to macromolecular targeted drugs remains a challenge [11]. NOAH study showed that 42% of patients treated with trastuzumab relapsed within 5 years [12]. Therefore, it is particularly important to seek targeted drugs with other mechanisms. Small molecule tyrosine kinase inhibitors (TKIs), as an alternative to HER2-targeted blockade, have the advantages of limiting multiple targets, possessing lower cardiac toxicity compared with monoclonal antibodies, and could be taken orally. It blocks HER2 signaling by competing with intracellular adenosine triphosphate (ATP), thereby preventing phosphorylation and changes in downstream molecular pathways. Therefore, TKIs may have some clinical advantages over monoclonal antibodies and may overcome some mechanisms of resistance to monoclonal antibodies, which brings more treatment options for patients with HER2-positive breast cancer [13]. Pyrotinib is an oral pan-ErbB inhibitor that irreversibly inhibits the tyrosine kinase activity of HER1 (epidermal growth factor receptor, EGFR), HER2, and HER4 [14]. It exhibits good tolerance and anti-tumor activity in advanced and metastatic HER2-positive breast cancer [15]. However, its efficacy in neoadjuvant therapy is still under exploration. Some trials of pyrotinib combined with other anti-tumor drugs in the neoadjuvant phase have been carried out [16–19]. However, there is still a lack of evidence to support the use of pyrotinib in the neoadjuvant phase. To further fill the gap and investigate the efficacy and safety of pyrotinib in patients receiving 4 cycles epirubicin and cyclophosphamide followed by 4 cycles of taxane and trastuzumab (EC-TH) regimen in the neoadjuvant phase, this study was therefore conducted.

The p53 is a tumor suppressor gene, which is considered the guardian of the genome. It preserves the integrity of the genome, in response to different stresses, by inducing cell cycle arrest, apoptosis, DNA repair, or metabolic adaptation [20–23]. The mutation of p53 sometimes causes the expression of a stable, yet transcriptionally deficient mutant-p53 protein. Expression of mutant p53 is associated with poor prognosis in human tumors and the development of aggressive tumors in mouse models [24]. Mutant-p53 increases cell growth, survival, and chemoresistance through a variety of mechanisms [25], for example by activating mitochondrial metabolism [26], which promotes cancer survival and resistance to different treatments [27, 28]. The p53 is the most frequently mutated gene in most types of human cancer, including breast cancer, and the p53 gene is mutated in 30–35% of invasive primary breast cancers [29]. Based on this high prevalence, mutant p53 might be expected to be a biomarker and a new therapeutic target for breast cancer. Besides, mutations in p53 are clonal, thus might be expected that clonal mutations would be better therapeutic targets for cancer treatment than sub-clonal or branching mutations [30, 31]. The p63 is the most ancient member of the p53 family of transcription factors. The human TP63 gene is located on chromosome 3q27–29. The p63 activity is critically involved in sustaining the proliferative potential and self-renewing capacity of mammary epithelial stem cells [32]. Studies have shown that p63-positive is associated with better overall survival of breast cancer [33]. A kind of isoform of p63 is known as ΔNp63. An important consequence of increased levels of ΔNp63 is that breast cancer stem cells (BCSCs) acquire resistance to aromatase inhibitors and taxanes. It will be crucial in the future to find a way to selectively target ΔNp63 or its downstream master regulators, such as PI3K or CD44v6. The inhibition of the PI3K/CD44v6 axis in combination with current anti-tumor therapies could be a very promising strategy to overcome primary tumor formation, metastasis, and relapses [34]. EGFR is a transmembrane cell receptor with tyrosine kinase activity, which is related to the proliferation, metastasis, invasion, angiogenesis, and inhibition of apoptosis of tumor cells. Research has shown that TKIs targeting specific EGFR mutations can significantly improve lung cancer patients’ outcomes and have become the preferred treatment option [35]. However, EGFR inhibitors have not achieved satisfactory clinical results in breast cancer. Inspired by this, we also explored the potential predictive value of p53, p63, and EGFR status for neoadjuvant therapy efficacy, as well as the potential of p53, p63, and EGFR mutations as new therapeutic targets for breast cancer.

## Methods

### Objectives

The main objectives of this study are as follows: (1) to evaluate the efficacy and safety of the EC-TH regimen in the neoadjuvant phase of HER2-positive breast cancer, and further fill the blank in the study of pyrotinib in the neoadjuvant phase. The total pathologic complete response (tpCR), breast pathologic complete response (bpCR), and overall response rate (ORR) were the main objective indicators used to evaluate the treatment efficacy. (2) To analyze the predictors of tpCR for HER2-positive breast cancer. (3) To explore the potential predictive value of p53, p63, and EGFR status in neoadjuvant therapy.

### Study design

Our study was a single-center prospective cohort study. The study conformed to the provisions of the Declaration of Helsinki (as revised in 2013) and informed consent was obtained from all individual participants. The sample size of the case group was determined by the number of patients with HER2-positive breast cancer who received therapy of pyrotinib combined with EC-TH (P + EC-TH) in the neoadjuvant phase in our center during the study period, and the sample size of the control group was determined by simple random sampling among all eligible patients. The ratio of the case group to the control group was approximately 1:1.5.

### Study objects

The subjects of this study were patients with early or locally advanced HER2-positive breast cancer who received neoadjuvant treatment followed by surgery in the breast surgery department of the First Affiliated Hospital of Zhengzhou University from July 2018 to July 2022.

### Inclusion and exclusion criteria

Of the 164 initially collected patients, 138 were included in the final analysis. Inclusion criteria are as follows: (1) females aged ≥ 18 years, with operable HER2-positive (immunohistochemically 3 + , or immunohistochemically 2 + but HER2 gene amplification in fluorescence in situ hybridization [FISH]) invasive breast cancers, clinical stage was diagnosed as II-III according to 8th edition American Joint Committee on Cancer (AJCC) tumor, node, metastasis (TNM) staging system, and the diagnosis of the clinical stage was based on breast ultrasonography, mammography, CT and MRI results. Histologic stage was diagnosed as II–III according to the World Health Organization (WHO) criteria. (2) Eastern Cooperative Oncology Group (ECOG) performance status scored 0–1. (3) At least one measurable lesion existed according to Response Evaluation Criteria in Solid Tumor (RECIST) version 1.1. (4) The function of the main organs was normal, such as the left ventricular injection fraction (LVEF) of cardiac ultrasound was ≥ 50%. (5) Life expectancy was more than 6 months. Exclusion criteria are as follows: (1) received any treatment for breast cancer before. (2) Inflammatory breast cancer (IBC), occult breast cancer, bilateral breast cancer, or distant metastasis. Abdominal and pelvic ultrasonography, chest CT, and cephalic MRI were routinely applied to identify breast cancer patients with distant metastasis. Further examinations were chosen according to the patients’ signs, symptoms, laboratory tests, and examination results. For example, single photon emission computed tomography (SPECT) was applied if the patients had ostealgia, pathological fractures, elevated alkaline phosphatase, or hypercalcemia. (3) Comorbidity of any other severe or uncontrolled systemic diseases. (4) Simultaneously suffered from other malignant tumors. (5) Pregnancy or lactation. (6) Neoadjuvant therapy was more or less than 8 cycles. (7) Incomplete clinical data.

### Treatment

Control group: 4-cycle epirubicin 100 mg/m^2^ (I.V.) + cyclophosphamide 600 mg/ m^2^ (I.V.), followed by 4-cycle paclitaxel 80 mg/ m^2^ (I.V.)/docetaxel 100 mg/m^2^ (I.V.) + trastuzumab 8 mg/kg (I.V.), and then 6 mg/kg (I.V.), each cycle lasted 21 days. Case group: The patients received 8 cycles of oral pyrotinib 400 mg/day additionally, reduction of pyrotinib to 320 mg/day or 240 mg/day due to adverse events (AEs) was allowed. Other treatment was the same as the control group. Individualized supportive treatments for different AEs were applied during chemotherapy when the corresponding AEs occurred. Suspended red blood cells (SRBCs) were transfused for severe anemia with hemoglobin below 60 g/L. Granulocyte colony-stimulating factor (G-CSF) was used to treat myelosuppression after chemotherapy. Oral or intravenous potassium supplementation was chosen for hypokalemia. Common hepatoprotective drugs like magnesium isoglycyrrhizinate were used for patients with abnormal liver function. Montmorillonite powder or loperamide was administered for diarrhea. Dexamethasone was used for allergic reactions. Vitamin supplementation was provided for hand-foot syndrome or stomatitis, etc. All patients underwent surgical treatment within 28 days after the final chemotherapy. Follow-up treatment plans for patients were based on best practices and patient preferences, such as postoperative radiotherapy for patients with axillary lymph node metastasis, endocrine therapy for hormone receptor (HR) positive patients, follow-up targeted therapy, etc.

### Efficacy evaluation

The RECIST version 1.1 was referred to evaluate the clinical efficacy of the case group and control group. (1) Complete Response (CR): Disappearance of all target lesions. Any pathological lymph nodes (whether target or non-target) must have a reduction in the short axis to < 10 mm. (2) Partial Response (PR): At least a 30% decrease in the sum of diameters of target lesions, taking as reference the baseline sum diameters. (3) Stable disease (SD): changes in the sum of the maximum diameter of target lesions were between PR and PD. (4) Progressive disease (PD): at least a 20% increase in the sum of diameters of target lesions, taking as reference the smallest sum on study, or a new lesion occurred. (5) Overall response rate (ORR): ORR = (CR + PR)/total lesions × 100%. (6) Total pCR (tpCR, ypT0/is N0): absence of invasive cancer components in the breast and axillary lymph nodes, and possible presence of carcinoma in situ components. (7) Breast pCR (bpCR): absence of invasive carcinoma in the primary breast tumor, and possible presence of carcinoma in situ components. The primary endpoint of our study was tpCR, while the secondary endpoints were bpCR, ORR, and AEs. AEs during the neoadjuvant therapy were evaluated using the National Cancer Institute Common Terminology Criteria for Adverse Event (NCI-CTCAE) version 5.0. The main data came from laboratory results, and a few subjective indicators came from telephone follow-ups. Physical examination and laboratory evaluation for patients were carried out before each chemotherapy. Breast ultrasonography and magnetic resonance imaging were rechecked every 2 cycles of chemotherapy to monitor target lesions. The pathological efficacy was based on routine pathological and immunohistochemical (IHC) results of samples before and after surgery. All medical imaging and pathological results have been double-confirmed by two professionals.

### p53, p63, and EGFR status

The routine pathological and IHC results were completed independently by two experienced pathologists. EGFR is mostly expressed in the cytomembrane and cytoplasm, cells with emerging brown-yellow or brown–red particles in the IHC were interpreted as positive cells, and the positive cell count > 5% under the microscope was interpreted as IHC positive. The p53 is mostly expressed in the nucleus, cells with emerging brown-yellow or brown–red particles in the IHC were interpreted as positive cells, and the positive cell count > 10% under the microscope was interpreted as IHC positive. The p63 is mostly expressed in the nucleus, cells with emerging red nuclei in the IHC were interpreted as positive cells, and the positive cell count > 10% under the microscope was interpreted as IHC positive.

### Statistical analysis

All statistical analyses were finished on IBM SPSS 26.0 (IBM Corp., Chicago, IL, USA). Continuous variables were expressed as the median (range), while categorical variables were expressed as a frequency (percentage). All the statistical tests were two-sided. *P* value < 0.05 was considered to have statistical significance. The chi-square test and Fisher’s exact test were used for the comparison of disordered data. The chi-square test was also used to compare tpCR, bpCR, ORR, and AEs differences between the case group and control group. Univariate logistic regression analysis was used to select predictors that were related to tpCR by a *P* value of 0.25 or less and multivariable logistic regression analysis was used to analyze these predetermined factors.

## Results

### Participants

**Fig. 1:**
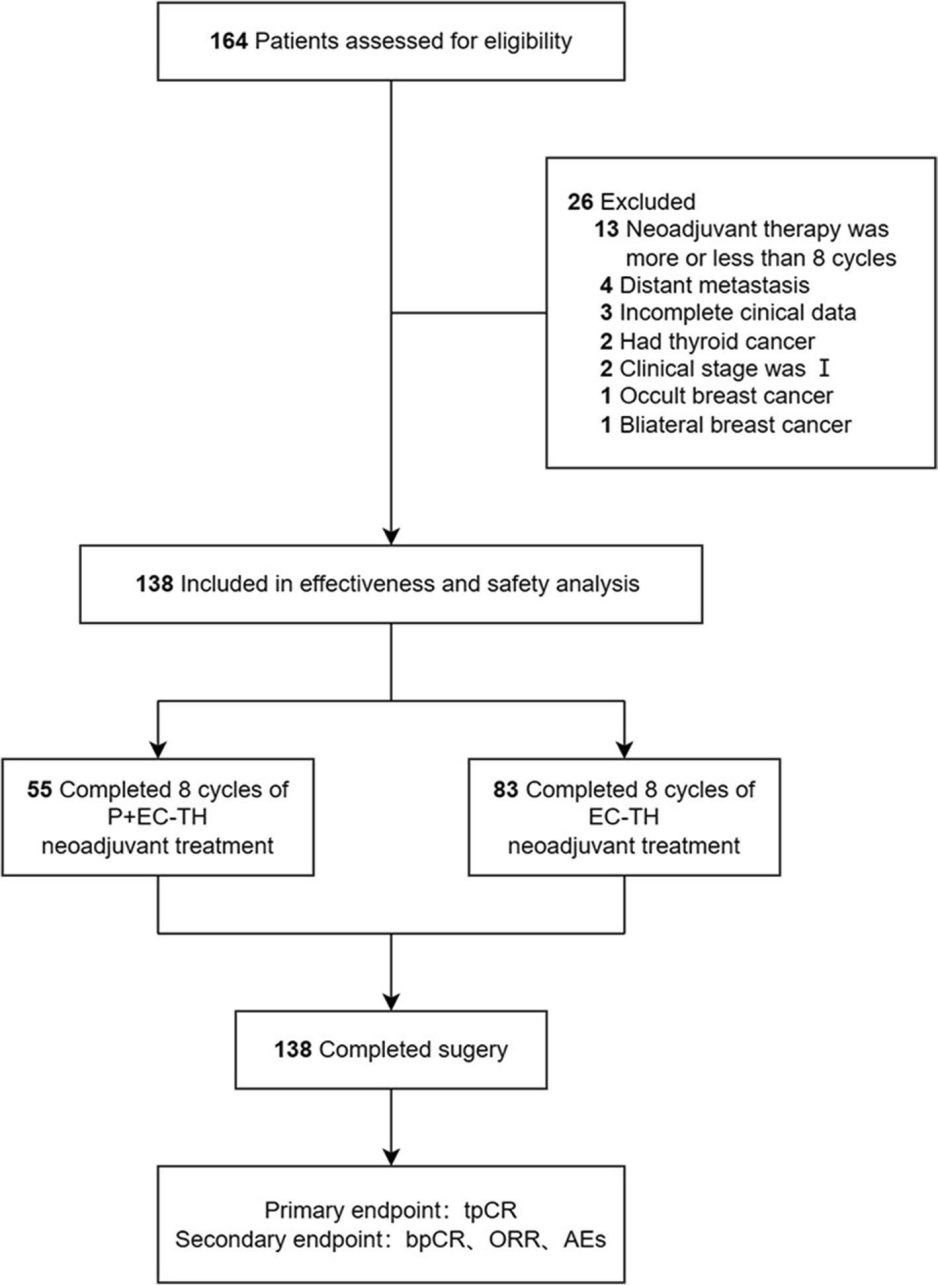
Study flowchart. P, pyrotinib; E, epirubicin; C, cyclophosphamide; T, paclitaxel/docetaxel; H, trastuzumab; tpCR, total pathologic complete response; bpCR, breast pathologic complete response; ORR, overall response rate; AEs, adverse events

According to the inclusion criteria, 164 patients with HER2-positive breast cancer who received neoadjuvant therapy in the Department of Breast Surgery of the First Affiliated Hospital of Zhengzhou University from July 2018 to July 2022 were collected. 13 patients’ neoadjuvant therapy was more or less than 8 cycles, 4 patients had distant metastasis, 2 patients were also found to have thyroid cancer, 2 patients’ clinical stage was I, 1 patient had occult breast cancer, 1 patient had bilateral breast cancer, and 3 patients lacked clinical data. Twenty-six patients were excluded according to the exclusion criteria. A total of 138 patients were included in the final study (Fig. 1). In this study, 55 patients in the case group received the P + EC-TH neoadjuvant therapy. The dose of pyrotinib was adjusted to 320 mg/day in 5 patients and 240 mg/day in 2 patients due to AEs. None of the other patients experienced drug reduction or discontinuation. In the control group, 83 patients received the EC-TH neoadjuvant therapy. A total of 102 patients were treated with paclitaxel (41 in the case group and 61 in the control group), and 36 with docetaxel (14 in the case group and 22 in the control group). All 138 patients underwent surgical treatment within 28 days after the final chemotherapy, of which 118 patients underwent modified radical mastectomy surgery, 6 patients underwent modified radical mastectomy and prosthesis reconstruction surgery, 3 patients underwent modified radical mastectomy and autologous latissimus dorsi reconstruction surgery, 7 patients underwent mastectomy and sentinel lymph node biopsy surgery, and 4 patients underwent breast-conserving and axillary lymph node dissection surgery. Participants were all female, with a median age of 48.5 years (range 20–67 years) and ECOG performance status were all 0–1. There were no significant differences in age, menopause status, ECOG performance status, clinical stage, histological stage, or molecular subtypes between the two groups (*P* > 0.05) (Table 1).

**Table 1:** Baseline characteristics

Characteristic	Total, *n*(%)	Patients, *n* (%)	
		Case group (*n* = 55)	Control group (*n* = 83)
Median/years	48.5(20–67)	46(20–66)	52(28–67)
Range/years			
< 60 years	128(92.75)	52(94.55)	76(91.57)
≥ 60 years	10(7.25)	3(5.45)	7(8.43)
Menopause status			
Premenopausal	83(60.14)	38(69.09)	45(54.22)
Postmenopausal	55(39.86)	17(30.91)	38(45.78)
ECOG performance status			
0	118(85.51)	47(85.45)	71(85.54)
1	20(14.49)	8(14.55)	12(14.46)
Primary tumor size			
T1	8(5.80)	3(5.45)	5(6.02)
T2	86(62.32)	32(58.18)	54(65.06)
T3	44(31.88)	20(36.36)	24(28.92)
Primary lymph node status			
N0	31(22.46)	13(23.64)	18(21.69)
N1	44(31.88)	19(34.55)	25(30.12)
N2	51(36.96)	20(36.36)	31(37.35)
N3	12(8.70)	3(5.45)	9(10.84)
Clinical stage			
II	62(44.93)	26(47.27)	36(43.37)
III	76(55.07)	29(52.73)	47(56.63)
Histologic stage			
II	99(71.74)	41(74.55)	58(69.88)
III	39(28.26)	14(25.45)	25(30.12)
ER			
Positive	63(45.65)	21(38.18)	42(50.60)
Negative	75(54.35)	34(61.82)	41(49.40)
PR			
Positive	54(39.13)	19(34.55)	35(42.17)
Negative	84(60.87)	36(65.45)	48(57.83)
AR			
Positive	117(84.78)	47(85.45)	70(84.34)
Negative	21(15.22)	8(14.55)	13(15.66)
Ki-67 levels			
< 14%	5(3.62)	2(3.64)	3(3.61)
≥ 14%	133(96.38)	53(96.36)	80(96.39)

*ECOG* Eastern Cooperative Oncology Group, *TNM* tumor, node, metastasis, *ER* estrogen receptor, *PR* progesterone receptor, *AR* androgen receptor. The positive threshold of ER and PR immunohistochemical staining is ≥ 1%

### Efficacy of case group and control group

After neoadjuvant therapy and surgery, 35(63.64%) of the 55 patients in the case group achieved tpCR, including 31.43% HR-positive (11/35), 68.57% HR-negative (24/35), 82.86% androgen receptor (AR) positive (29/35), and 17.14% AR-negative (6/35). bpCR was achieved in 38 patients (69.09%), and ORR was 100% (55/55). Of the 83 patients in the control group, 33(39.76%) achieved tpCR, including 45.45% HR-positive (15/33), 54.55% HR-negative (18/33), 72.73% AR-positive (24/33), and 27.27% AR-negative (9/33). bpCR was achieved in 37 patients (44.58%). The clinical efficacy in 4 patients was evaluated as SD, and ORR was 95.18% (79/83).

The tpCR in the case group was significantly higher than that in the control group (63.64% vs 39.76%, *P* = 0.006), and in the case of bpCR, the case group was significantly higher than the control group (69.09% vs 44.58%, *P* = 0.005) as well. There was no significant difference in ORR between the two groups (100.0% vs. 95.18%, *P* = 0.257) (Table 2). There was no significant difference in tpCR and bpCR rates between patients treated with paclitaxel and those treated with docetaxel (*P* > 0.05). As of the May 2023 data cutoff date, the median follow-up time was 23 months (range 4–52 months). In the case group, 3 patients developed distant metastasis, including 1 lung metastasis, 1 bone metastasis, and 1 brain metastasis. No death occurred during the follow-up (median follow-up time was 15 months). The metastasis rate was 5.45% (3/55), the PFS was 15.15 ± 7.66 months, the 1-year recurrence and metastasis rate were 1.82% (1/55), and the 1-year overall survival rate was 100.00%. In the control group, 1 patient developed regional metastasis of supraclavicular lymph node, 8 patients developed distant metastasis, including 2 patients with lung metastasis, 2 with brain metastasis, and 4 patients with multiple site recurrence and metastasis. The metastasis rate was 10.84% (9/83), PFS was 28.27 ± 12.75 months (median follow-up time was 30 months), and 2 patients died of cancer recurrence and metastasis. The 1-year recurrence and metastasis rate were 6.02% (5/83), and 1-year overall survival rate was 100%. There were no significant differences in 1-year recurrence and metastasis rate and 1-year overall survival rate between the case group and control group (*P* > 0.05). Longer follow-up is needed to compare the differences in long-term efficacy between the two groups.

**Table 2:** Efficacy of case group and control group

Groups	Cases	tpCR,*n*(%)	bpCR, *n*(%)	ORR, *n*(%)	CR, *n*(%)	PR, *n*(%)	SD, *n*(%)
Total	138	68(49.28)	75(54.35)	134(97.10)	29(21.01)	105(76.09)	4(2.90)
Case	55	35(63.64)	38(69.09)	55(100.00)	15(27.27)	40(72.73)	0(0.00)
Control	83	33(39.76)	37(44.58)	79(95.18)	14(16.87)	65(78.31)	4(4.82)
χ2		7.545	8.011	1.286	2.158	0.567	1.286
P		0.006	0.005	0.257	0.142	0.451	0.257

*Case group* Pyrotinib + EC-TH, *Control group* EC-TH; *tpCR* total pathologic complete response, *bpCR* breast pathologic complete response, *ORR* overall response rate, *CR* complete response, *PR* partial response, *SD* stable disease

### Factors influencing tpCR

The tpCR was associated with the status of estrogen receptor (ER), progesterone receptor (PR), and AR, and the patients with any negative ER, PR, AR, or combined were more likely to achieve tpCR than those with positive results (*P* < 0.05) (Table 3). At the same time, the patients with either negative PR or AR were more likely to achieve bpCR than those with positive results (*P* < 0.05).

**Table 3:** Efficacy of diverse hormone receptor and androgen receptor status

Characteristics	tpCR, *n*(%)	Non-tpCR, *n*(%)	*χ*2	*P*	bpCR, *n*(%)	Non-bpCR, *n*(%)	*χ*2	*P*
ER								
Positive (*n* = 63)	23(33.82)	40(57.14)	7.560	0.006	29(38.67)	34(53.97)	3.231	0.072
Negative (*n* = 75)	45(66.18)	30(42.86)			46(61.33)	29(46.03)		
PR								
Positive (*n* = 54)	18(26.47)	36(51.43)	9.021	0.003	22(29.33)	32(50.79)	6.620	0.010
Negative (*n* = 84)	50(73.53)	34(48.57)			53(70.67)	31(49.21)		
HR								
ER and/or PR positive (*n* = 69)	26(38.24)	43(61.43)	7.422	0.006	32(42.67)	37(58.73)	3.534	0.060
ER and PR negative (*n* = 69)	42(61.76)	27(38.57)			43(57.33)	26(41.27)		
AR								
Positive (*n* = 117)	53(77.94)	64(91.43)	4.863	0.027	59(78.67)	58(92.06)	4.763	0.029
Negative (*n* = 21)	15(22.06)	6(8.57)			16(21.33)	5(7.94)		

*tpCR* total pathologic complete response, *bpCR* breast pathologic complete response. *ER* estrogen receptor; progesterone receptor, *HR* hormone receptor, *AR* androgen receptor

Of the 68 patients who achieved tpCR, 63 (92.65%) patients were HER2 3 + as detected by IHC, and 5 patients (7.35%) were HER2 2 + , with HER2 gene amplification in FISH (HER2 2 + /FISH +). Of the 70 patients who didn't achieve tpCR, 49 (70.00%) were HER2 3 + , and 21 (30.00%) were HER2 2 + /FISH + . HER2 3 + patients were more likely to achieve tpCR than HER2 2 + /FISH + patients (*P* = 0.001).

**Fig. 2:**
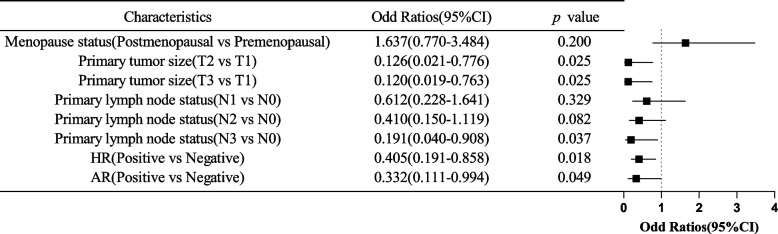
Logistic regression analysis of tpCR predictive factors

The independent predictors of tpCR were further analyzed, and the patient characteristics were included in logistic regression analysis. The results showed that the tumor stage being T1, the lymph node stage being N0, HR-negative, and AR-negative were the independent predictors of tpCR (Fig. 2). Patients in T1 stage were more likely to achieve tpCR than those in T2 stage (*P* = 0.025, OR = 0.126, 95%CI 0.021–0.776), and T1 patients were also more likely to achieve tpCR than T3 patients (*P* = 0.025, OR = 0.120, 95%CI 0.019–0.763). Patients in the N0 stage were more likely to achieve tpCR than those in the N3 stage (*P* = 0.037, OR = 0.191, 95%CI 0.040–0.908). HR-negative patients were more likely to achieve tpCR than HR-positive patients (*P* = 0.018, OR = 0.405, 95%CI 0.191–0.858), and AR-negative patients were more likely to achieve tpCR than AR-positive patients (*P* = 0.049, OR = 0.332, 95%CI 0.111–0.994). Our study was not able to demonstrate whether other baseline characteristics were independent predictors of tpCR or not.

### Predictive value of p53 and p63 status for efficacy

Of the 138 patients in this study, 112 had their p53 status detected before neoadjuvant therapy. p53 was positive in 63 cases (56.25%) and negative in 49 cases (43.75%). p53 status had significant predictive value for tpCR and bpCR, and patients with positive p53 were more likely to achieve tpCR and bpCR than those with negative results (Table 4), but there was no significant predictive value for clinical efficacy (*P* > 0.05). p63 expression status was detected in 132 patients before the neoadjuvant therapy, of which 14 (10.61%) were positive and 118 (89.39%) were negative. p63 expression status had no significant predictive value for pathological and clinical efficacy (*P* > 0.05).

**Table 4:** Efficacy of diverse p53 expression status

p53	Pathological efficacy		Clinical efficacy			Total, *n*(%)
	tpCR, *n*(%)	bpCR, *n*(%)	CR, *n*(%)	PR, *n*(%)	SD, *n*(%)	
Positive	36(66.67)	39(65.00)	14(56.00)	47(55.95)	2(66.67)	63(56.25)
Negative	18(33.33)	21(35.00)	11(44.00)	37(44.05)	1(33.33)	49(43.75)
χ2	4.598	4.021	0.001	0.012	0.000	
P	0.032	0.045	0.977	0.912	1.000	

*tpCR* total pathologic complete response, *bpCR* breast pathologic complete response, *CR* complete response, *PR* partial response, *SD* stable disease

### Predictive value of EGFR status for efficacy

Of the 138 patients in this study, 110 had their EGFR expression status detected before neoadjuvant therapy. EGFR was positive in 48 cases (43.64%) and negative in 62 cases (56.36%). EGFR status had no significant predictive value for pathological and clinical efficacy (*P* > 0.05). However, we observed that in EGFR-positive patients, the tpCR rate was as high as 76.47% (13/17) in the case group, while the tpCR rate was only 45.16% (14/31) in the control group, and the difference in tpCR rate between the two groups was statistically significant (*P* < 0.05). However, in EGFR-negative patients, the tpCR rate was 58.06% (18/31) in the case group, while the tpCR rate was 35.48% (11/31) in the control group, and there was no significant difference in tpCR rates between the two groups (*P* > 0.05). This suggests that EGFR-positive patients may benefit more from using pyrotinib during the neoadjuvant period.

### Adverse events

The most common AE in the case group was diarrhea (Table 5), with an incidence of 96.36% (53/55), among which grade 3 diarrhea occurred at 25.45% (14/55), and grade 4 diarrhea was not observed. The second most common AEs were anemia and alopecia, both with an incidence of 87.27% (48/55). In the control group, the most common AE was alopecia, with an incidence of 92.77% (77/83), followed by anemia, with an incidence of 75.90% (63/83). The incidences of hypokalemia and diarrhea in the case group were higher than those in the control group (*P* < 0.05), and there were no statistically significant differences in other AEs between the two groups (*P* > 0.05). Two patients in the control group with hemoglobin levels below 60 g/L were each transfused with 2 units of SRBCs once. Subsequent testing the day after the transfusion revealed an increase in hemoglobin levels above 60 g/L. Grade 4 AEs occurred only in 4 patients in the case group, all of which were hypokalemia, considered to be related to diarrhea caused by pyrotinib, and all of them were improved after potassium supplement and antidiarrheal therapy. There was no significant difference in the incidence of AEs between patients treated with paclitaxel and those treated with docetaxel (*P* > 0.05). All AEs were tolerated, no patient discontinued treatment due to AEs and no treatment-related death occurred.

**Table 5:** Adverse events of the case group and control group

AEs	Case group, *n*(%)	Control group, *n*(%)	*χ*2	*P*
Anemia				
Any grade	48(87.27)	63(75.90)	2.717	0.099
≥ 3	4(7.27)	6(7.23)	0.000	1.000
WBC decreased				
Any grade	22(40.00)	32(38.55)	0.029	0.865
≥ 3	2(3.64)	4(4.82)	0.000	1.000
Neutropenia				
Any grade	17(30.91)	25(30.12)	0.010	0.921
≥ 3	2(3.64)	6(7.23)	0.262	0.609
Thrombocytopenia				
Any grade	2(3.64)	6(7.23)	0.262	0.609
≥ 3	0(0.00)	2(2.41)	–	0.517
Hypokalemia				
Any grade	17(30.91)	2(2.41)	22.630	0.000
≥ 3	7(12.73)	0(0.00)	8.642	0.003
ALT increased				
Any grade	11(20.00)	11(13.25)	1.124	0.289
≥ 3	4(7.27)	2(2.41)	0.894	0.345
AST increased				
Any grade	4(7.27)	4(4.82)	0.054	0.817
≥ 3	2(3.64)	1(1.20)	0.132	0.717
Urea nitrogen increased				
Any grade	7(12.73)	7(8.43)	0.669	0.413
≥ 3	0(0.00)	0(0.00)	–	–
Diarrhea				
Any grade	53(96.36)	22(26.51)	65.065	0.000
≥ 3	14(25.45)	1(1.20)	20.079	0.000
Nausea				
Any grade	36(65.45)	55(66.27)	0.010	0.922
≥ 3	0(0.00)	0(0.00)	–	–
Vomiting				
Any grade	28(50.91)	41(49.40)	0.030	0.862
≥ 3	3(5.45)	6(7.23)	0.004	0.951
Asthenia				
Any grade	31(56.36)	37(44.58)	1.838	0.175
≥ 3	0(0.00)	0(0.00)	–	–
Rash				
Any grade	13(23.64)	15(18.07)	0.633	0.426
≥ 3	0(0.00)	2(2.41)	–	0.517
Pigmentation				
Any grade	19(34.55)	29(34.94)	0.002	0.962
≥ 3	0(0.00)	0(0.00)	–	–
Hand-foot syndrome				
Any grade	8(14.55)	15(18.07)	0.296	0.586
≥ 3	0(0.00)	1(1.20)	–	1.000
Alopecia				
Any grade	48(87.27)	77(92.77)	1.172	0.279
≥ 3	0(0.00)	0(0.00)	–	–
Stomatitis				
Any grade	18(32.73)	23(27.71)	0.399	0.528
≥ 3	4(7.27)	5(6.02)	0.000	1.000

## Discussion

HER2 is a transmembrane tyrosine kinase (TK) receptor, whose overexpression often leads to adverse clinical outcomes in breast cancer and reduces sensitivity to various chemotherapy drugs and hormones. The emergence of anti-HER2 targeting drugs has greatly improved the prognosis of patients with HER2-positive breast cancer. Trastuzumab, developed in 1988, is the first humanized monoclonal antibody drug for the treatment of HER2-positive breast cancer, which can selectively bind to the outer cell site of HER2 and inhibit the proliferation of tumor cells [36]. TKIs, as small molecular compounds, have the ability to cross the blood–brain barrier. Due to their different modes of action, TKIs may be able to overcome some resistance mechanisms of trastuzumab, showing potential therapeutic advantages [37]. Currently, TKIs in clinical use include lapatinib, neratinib, and pyrotinib. A multicenter NeoALTTO study from 23 countries confirmed that neoadjuvant therapy with lapatinib combined with trastuzumab induced a higher pCR rate than trastuzumab alone (*P* = 0.0001) [38]. CALGB 40601 study [39], CHER-LOB study [40], and TRIO-US B07 study [41] also verified that lapatinib and trastuzumab combined with chemotherapy could increase the pCR rate. These studies all confirmed that TKIs lapatinib and trastuzumab have complementary mechanisms of action and synergistic antitumor activity in HER2 overexpression breast cancer. Subsequently, the PHOEBE study compared the efficacy of pyrotinib and lapatinib in metastatic breast cancer, and the results showed that compared with lapatinib plus capecitabine, pyrotinib plus capecitabine significantly improved PFS, and the toxicity was manageable. Pyrotinib is a new irreversible TKI, which has a strong irreversible inhibitory effect on both EGFR and HER2 [42]. Pyrotinib can overcome the resistance problem of macromolecular targeted drugs, and its efficacy has been verified in patients with metastatic or advanced breast cancer. Ma et al. revealed that pyrotinib showed good antitumor activity in patients with HER2-positive metastatic breast cancer and determined the maximum tolerated dose of pyrotinib to be 400 mg [43]. The PERMEATE study was the first to point out that pyrotinib combined with capecitabine has good antitumor activity against brain metastasis, especially in people who have not received radiation therapy [44]. Nevertheless, the efficacy of pyrotinib in the neoadjuvant phase remains to be explored, and some studies have already been conducted on the use of chemotherapy combined with pyrotinib in the neoadjuvant phase. Xuhong et al. reported for the first time that the tpCR rate of the P + EC-TH neoadjuvant regimen in operable or locally advanced HER2-positive breast cancer was 73.7% (95% CI 48.8–90.9), which was about twice as high as that of the EC-TH neoadjuvant regimen reported in other trials, and the AEs were tolerable. Short-term follow-up showed no recurrence or metastasis [17]. Zhong et al. evaluated the efficacy and safety of neoadjuvant pyrotinib plus trastuzumab and albumin-bound paclitaxel therapy in patients with HER2-positive breast cancer, with an overall pCR rate of 57.1% (12/21). At the end of neoadjuvant therapy, ORR reached 100% (21/21) [18]. In the Panphila study, 55.1% patients (38/69) achieved pCR in the modified intention-to-treat population who received neoadjuvant pyrotinib plus trastuzumab and chemotherapy [16]. These studies above have revealed the efficacy of pyrotinib in the neoadjuvant phase. However, they were limited by their small sample size or the lack of a control group. Subsequently, the PHEDRA study conducted a randomized phase 3 trial of neoadjuvant pyrotinib, trastuzumab, and docetaxel in HER2-positive breast cancer, and the results confirmed that the tpCR rate in the pyrotinib group was 41.0% (95% CI 34.0–48.4), while 22.0% (95% CI 16.6–28.7) in the placebo group (difference 19.0% [95% CI 9.5–28.4]; Unilateral *P* < 0.0001) [19]. The efficacy of neoadjuvant pyrotinib plus trastuzumab dual-target therapy combined with chemotherapy has been gradually verified. In the 2022 version of the Chinese Society of Clinical Oncology breast cancer guideline and The National Comprehensive Cancer Network guideline, anthracycline/cyclophosphamide followed by taxane/trastuzumab [AC-TH] is the recommended neoadjuvant therapy with obvious curative effect for HER2-positive breast cancer patients [5, 6]. Anthracyclines are cardiotoxic and can cause congestive heart failure when combined with trastuzumab. Sequential regimens can reduce the occurrence of heart failure. However, the efficacy and safety of pyrotinib combined with this regimen remain to be explored. Inspired by this, to explore the efficacy and safety of the P + EC-TH regimen, and provide data support for the use of pyrotinib in the neoadjuvant phase, we conducted this study.

Our study was a prospective cohort study. In our study, the tpCR rate in the pyrotinib group reached 63.64%, which was significantly higher than 39.76% in the control group. The tpCR rate in our pyrotinib group was higher than 41.0% in the PHEDRA study, 57.1% reported by Zhong et al., and 55.1% in the Panphila study, which may be related to the fact that the neoadjuvant therapy cycles in the studies above were only 4 or 6, and only taxane was used for chemotherapy. In our study, there were a total of 8 cycles of neoadjuvant therapy, and epirubicin plus cyclophosphamide followed by taxane were used as chemotherapy drugs. However, the tpCR rates in the above two studies were still higher than that in our control group without the use of pyrotinib, which suggests that the combinations of macromolecular targeted drugs and TKIs, which possess different mechanisms, may be more effective than the combinations of multiple chemotherapy drugs. A 10-year study showed no significant difference in efficacy between AC-TH and TCH, but comparing with AC-T, the AC-TH regimen has received continued significant benefits for DFS [45]. This suggests that future efforts in the comprehensive treatment of HER2-positive breast cancer should focus on exploring optimal targeted drug regimens.

Xuhong et al. reported that the tpCR rate of the P + EC-TH neoadjuvant regimen was 73.7%. Compared with their study, the low tpCR rate in our case group may be attributed to the fact that 7 people underwent dose adjustment due to AEs of pyrotinib. Nevertheless, the tpCR rates of the P + EC-TH neoadjuvant regimen in our study and Xuhong's study were about twice as high as that of the EC-TH neoadjuvant regimen reported in other trials, suggesting that patients with HER2-positive breast cancer treated with EC-TH neoadjuvant regimen can use pyrotinib simultaneously, obtaining a higher tpCR rate and better prognosis. However, our study did not compare the difference in efficacy between traditional trastuzumab plus pertuzumab versus trastuzumab plus pyrotinib. Which targeted therapy regimen is the most beneficial to patients during the neoadjuvant phase still needs to be explored in the future.

ER and PR status are indicators of molecular subtypes of breast cancer. Generally, HR-positive breast cancer has low malignancy and a good prognosis. Rouzier demonstrated by using a gene expression profile that patients with HR-negative were more likely to achieve pCR than those with HR-positive [46]. The study of Chen also verified this conclusion [47]. Patients with negative ER and PR were more likely to achieve pCR (*P* < 0.001) in Colleoni's study, and the pCR rate of patients with HR-negative was 4.22 times that of patients with HR-positive [48]. Our study also verified the conclusion reported in the above studies that ER and PR-negative patients were more likely to achieve tpCR than positive patients. The possible reasons are that HR-positive patients are not sensitive to chemotherapy, or HR-negative breast cancer is characterized by high expression of proliferative cluster genes [49], or HER2-positive and HR-negative tumors are highly dependent on the HER2 gene and therefore show a good response to anti-HER2-targeted therapy. HR-positive patients have a lower tpCR rate than negative patients, which suggests that HR-positive patients can be treated with endocrine therapy combined with chemotherapy in the neoadjuvant stage to achieve better efficacy.

AR belongs to the nuclear steroid hormone receptor family and acts as an intracellular transcription factor. AR is emerging as an important factor in the pathogenesis of breast cancer and may be a new marker and a potential therapeutic target among AR-positive breast cancer patients. Further studies have associated AR with better overall survival (*P* = 0.04), but also with lower rates of pCR to neoadjuvant chemotherapy [50]. Our study showed that AR-negative patients were more likely to achieve tpCR (*P* = 0.027) and bpCR (*P* = 0.029), which is consistent with the conclusions of the above studies. Therefore, the AR inhibitors approved for prostate cancer treatment could constitute a therapeutic tool for breast cancer, and become a new direction for endocrine therapy of breast cancer. However, there is a lack of relevant research at present, which is worth further exploration in the future.

Many studies have shown that TP53 mutant breast cancer has a significantly higher pCR rate during neoadjuvant therapy compared to wild-type breast cancer. Our study also proved that breast cancer patients with positive p53 expression detected by IHC were more likely to achieve tpCR (*P* = 0.032) and bpCR (*P* = 0.045) than those with negative p53 expression. However, in the EORTC 10994/BIG 1–00 trial, the presence of p53 mutations was found to be predictive of poor outcomes in patients treated with either anthracycline or taxane-based regimen [51], which may be caused by poor tumor differentiation and low ER expression in p53-positive patients. The correlation between p53 expression and NAC efficacy is controversial, possibly due to the inaccuracy of p53 status detected by IHC, and DNA sequencing is considered the gold standard for detecting TP53 gene mutations. The p53 can be used not only as a judgment factor of efficacy but also as a target for new targeted drugs to repair the regulation of mutated p53 on tumor cells and exert the anti-tumor effect better.

In this study, p63 expression status had no significant predictive value for pathological and clinical efficacy (*P* > 0.05). However, previous studies have found that compared with p63-negative patients, patients with positive p63 before chemotherapy can achieve a higher pCR rate [52]. A larger sample is needed to determine the predictive value of p63 for neoadjuvant efficacy in breast cancer and its potential as a new therapeutic target.

The expression status of EGFR had no significant predictive value for either pathological or clinical efficacy (*P* > 0.05), which may be caused by insufficient sample size. However, we observed that in EGFR-positive patients, the tpCR rate in the case group was significantly higher than that in the control group (76.47%vs. 45.16%, *P* < 0.05). Among EGFR-negative patients, there was no significant difference in tpCR rates between the two groups (58.06%vs. 35.48%, *P* > 0.05). The reason for this phenomenon may be that pyrotinib inhibits EGFR irreversibly, so EGFR-positive people are more likely to benefit from the use of pyrotinib during neoadjuvant therapy. Whether EGFR expression status can be used as a basis for screening the patients who are more likely to benefit from the use of pyrotinib is also worth further exploration in the future.

The overall toxicity of the case group was acceptable. The incidences of hypokalemia and diarrhea in the case group were higher than those in the control group, and there was no significant difference in other AEs between the two groups. The occurrence of hypokalemia was considered to be related to severe diarrhea, which was improved after potassium supplementation and anti-diarrhea treatment. Pyrotinib-induced diarrhea was the most common AE in the case group, with an incidence of 96.36%, which is consistent with previous reports (96.36%vs. 90–100%). The incidence of grade 3 diarrhea was slightly lower than previous results (25.45%vs. 28.6–45%) [16–19]. The absence of carboplatin during chemotherapy and the use of antidiarrheal interventions such as loperamide at the onset of diarrhea may be the reason for the low incidence of grade 3 diarrhea. No grade 4 diarrhea was observed.

Admittedly, there are some limitations in this study. This is a single-center study and only represents women in Central China, which may lead to a certain degree of bias. In addition, long-term survival data could not be observed due to the short follow-up time. Our conclusions still need to be verified in large-scale clinical trials, and the long-term efficacy of the neoadjuvant P + EC-TH regimen needs to be explored. Nevertheless, our study provides a factual basis for the application of pyrotinib in the neoadjuvant stage. This study verified the efficacy and safety of the P + EC-TH regimen and provides data support for this regimen.

## Conclusion

In conclusion, the neoadjuvant P + EC-TH regimen showed promising clinical benefits and acceptable safety in patients with HER2-positive breast cancer. TKIs combined with macromolecular targeted drugs and chemotherapy may be the main treatment method in the neoadjuvant phase for HER2-positive breast cancer patients in the future, and HER2-positive breast cancer patients with EGFR-positive may benefit more from the use of pyrotinib. The efficacy of AR inhibitors in AR-positive breast cancer patients deserves further study, and the targeted drugs for p53 also deserve further exploration. These conclusions still need to be further verified in large-scale clinical trials and long-term follow-ups.

## Data Availability

The datasets used or analyzed during the current study are available from the corresponding author on reasonable request.
